# Downregulation of ASPP2 in pancreatic cancer cells contributes to increased resistance to gemcitabine through autophagy activation

**DOI:** 10.1186/s12943-015-0447-5

**Published:** 2015-10-05

**Authors:** Bin Song, Qi Bian, Yi-Jie Zhang, Cheng-Hao Shao, Gang Li, An-An Liu, Wei Jing, Rui Liu, Ying-Qi Zhou, Gang Jin, Xian-Gui Hu

**Affiliations:** Department of General Surgery, Changhai Hospital, Second Military Medical University, Shanghai, China; Department of Nephrology, Changhai Hospital, Second Military Medical University, Changhai Road No.168, Shanghai, China

**Keywords:** Pancreatic cancer, ASSP2, Proliferation, Autophagy, Drug resistance, Gemcitabine

## Abstract

**Background:**

Apoptosis-stimulating of p53 protein 2 (ASPP2) is one of the ASPP family members and it has been reported to be associated with human cancer. However, the role of it in pancreatic cancer is still not clear.

**Methods:**

We analyzed the expression level of ASPP2 in cancer tissue samples with RT-qPCR, Western Blotting assay and immunohistochemistry staining. We studied the biological function of ASPP2 and its mechanism with gene overexpression and gene silencing technologies. We determined the sensitivity of pancreatic cells with differential ASPP2 level to gemcitabine and whether autophagy inhibition affected the gemcitabine resistance, both in vitro and in vivo.

**Results:**

Expression of ASPP2 was downregulated in cancerous tissues in comparison with para-cancerous tissues. ASPP2 expression was linked to clinical outcomes in patients and down-regulation of ASPP2 increased cell proliferation, autophagic flux, the activity of AMP Kinase of pancreatic cancer cells and vice versa. Knockdown of ASPP2 results in increased resistance to gemcitabine, which was attributed to the enhanced autophagy.

**Conclusions:**

ASSP2 expression is lower in cancerous tissues and decreased ASPP2 lead to higher cancer cells proliferation and autophagic flux, which contribute to the gemcitabine resistance.

**Electronic supplementary material:**

The online version of this article (doi:10.1186/s12943-015-0447-5) contains supplementary material, which is available to authorized users.

## Background

Pancreatic cancer (PC) is the fourth leading cause of cancer-related deaths in the world with 5-year survival rate of 4 %.[1, 2] Currently, the main therapeutic options include surgical resection, radiation therapy, chemotherapy and immunotherapy.[3] However, in most cases after surgery treatment, the tumor recurs within 1–2 years and patients develop metastasis. [4] In case of patients with inoperable PC, the standard treatment is chemotherapy. In this group, the survival of the patients is just increased by a dismal 5 weeks.[3, 4] The poor outcome of chemotherapy is partly due to the drug-resistant phenotype of PC cells.[5–9] however, the mechanism is not fully elucidated. Thus, failure of effective chemotherapy results in high mortality in PC patients, which the importance of understanding the mechanism of drug resistance and developing strategies that would improve the outcome of chemotherapy.

ASPP2 is a pro-apoptotic regulator that belongs to ASPP family.[10] The expression of ASPP2 is frequently suppressed in many cancers in relation to enhanced apoptosis through the binding to p53 for transcriptional transactivation.[11–15] Surprisingly, a number of ASPP2 binding partners that are involved in biological pathways other than apoptosis have also been identified,[10] suggesting that ASPP2 function is far more complex than simply enhancing p53-mediated apoptosis.

Autophagy acts as a survival mechanism under conditions of stress, maintaining cellular integrity by regenerating metabolic precursors and clearing subcellular debris [16]. Autophagy is wildly involved in the tumorous development and drug resistance.[17–20] A recent study has found that ASPP2 inhibits RAS-induced autophagy by preventing ATG16/ATG5/ATG12 formation and induces autophagic apoptosis by releasing Beclin-1 from cytoplasmic Bcl-2-Beclin-1 complexes in hepatoma cells.[21, 22] However, the function and detailed mechanism of ASPP2 which regulate autophagy is still not clear, especially in pancreatic cancer. In this study, we investigated the role and mechanism of ASPP2 for pancreatic cancer drug resistance.

Our data suggest that ASPP2 express lower in pancreatic cancer cells in comparison with para-cancer cells and decrease of ASPP2 expression is also linked to poor clinical outcomes in patients. Additionally, the decreased expression of ASPP2 can lead to enhanced autophagy through AMP kinase pathway in pancreatic cancer and resistance to gemcitabine in vitro and in vivo. Furthermore, the anticancer activity of ASPP2 in pancreatic cancer is partially due to its regulation on autophagy. Our study highlights ASPP2 and autophagy can be targeted for improvement of the efficiency of gemcitabine treatment and development of novel anti-pancreatic cancer drugs.

## Results

### ASSP2 expression is lower in cancerous tissues and predicts a poor prognosis

**Fig. 1:**
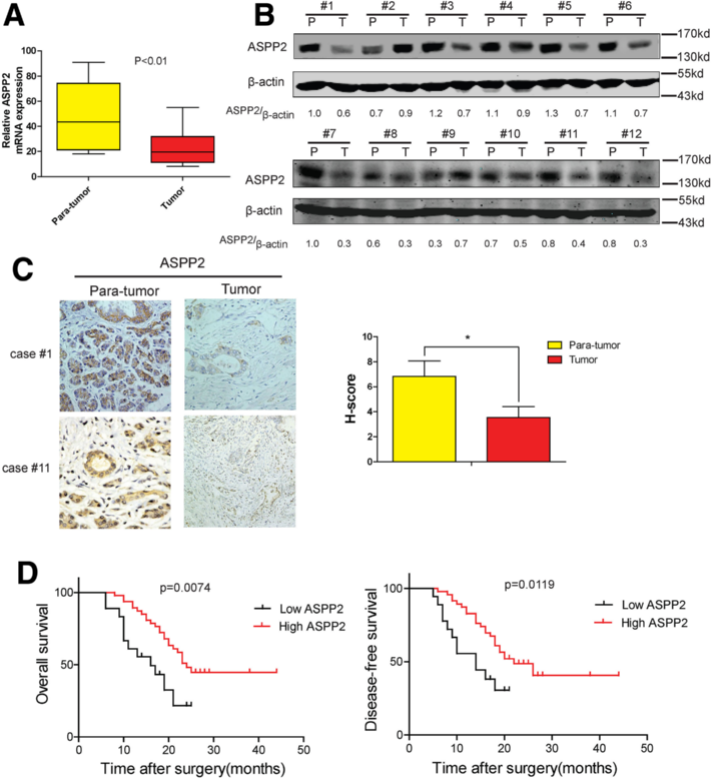
Downregulation of ASPP2 in Pancreatic cancer tissues. **a** mRNA from pancreatic cancer tissues and para-cancerous tissues was extracted and RT–qPCR analysis was performed to determine the expression of ASPP2; **b** Pancreatic cancer tissues and para-cancerous tissues from 12 patients were collected and Western Blot assay was performed to analyze the ASPP2 expression; **c** 65 Pancreatic cancer tissues and para-cancerous tissues were analyzed by immunohistochemical method to determine ASPP2 expression level. Representative samples were shown. The bar graph shows the statistical analysis of ASPP2 in pancreatic cancer tissues and para-cancerous tissues. **d** The disease-free and overall survival rates of patients of **c** were compared between the low-ASPP2 and high-ASPP2 groups. Data represent the mean ± SD of three independent experiments (*P < 0.05, **P < 0.01)

ASPP2 plays a key role in apoptosis regulation in the intrinsic and extrinsic apoptotic pathways [23, 24] and widely expressed in many human tissues.[25] The expression of ASPP2 can be enhanced in response to DNA damage,[13] and was down-regulated in both metastatic and invasive cells as compared to normal breast epithelium.[26] To exam the levels of expression of ASSP2 in the pancreatic cancer tissues and para-cancerous tissues, we determined ASSP2 expression level with RT-qPCR assay or immunoblotting assay, the ASSP2 expression level was less abundant in pancreatic cancer tissues compared with para-cancerous tissues (Fig. 1a and b). We also analyzed the patients’ samples with IHC assay (Table 1). As presented in Fig. 1c (left panel, 1# and 11# samples as the representative samples), the results suggest that ASSP2 protein expression was much lower in cancerous tissues (Fig. 1c, right panel). Furthermore, the ASPP2 low expression patients were found to exhibit high Histopathological grade (Table 1). More importantly, significant differences in both overall survival and disease-free survival were found among ASPP2 low/high groups (Fig. 1d). Taken together, all the data suggest that ASSP2 expression is lower in cancerous tissues than papa-cancerous tissues and it could be an independent prognostic predictor for pancreatic cancer.

**Table 1:** Correlation between ASPP2 expression levels and clinicopathological factors in PC (*n*=65)

	No. of patients	ASPP2 expression		*p*-value
		Low expression *n*=47	High expression *n*=18	
Age (years) Mean±SD	65	54.8+13.2	59.1+11.2	0.362
Gender				
Male	44	35	9	0.061
Female	21	12	9	
Histopathological grade				
G1	18	17	1	0.013
G2,3	47	30	17	
Depth of invasion				
T1, 2	41	27	14	0.133
T3, 4	24	20	4	
Lymph node metastasis				
Negative	33	23	10	0.639
Positive	32	24	8	
Pathologic stage				
I, II	59	44	15	0.203
III, IV	6	3	3	

Abbreviations: *PC* pancreatic cancer

Patients with PC were divided into ASPP2 “High” group (whose density was higher than the median) and “Low” group (whose density was lower than the median). The patient and disease profiles in each group were compared

### ASPP2 inhibit proliferation of pancreatic cancer cell

**Fig. 2:**
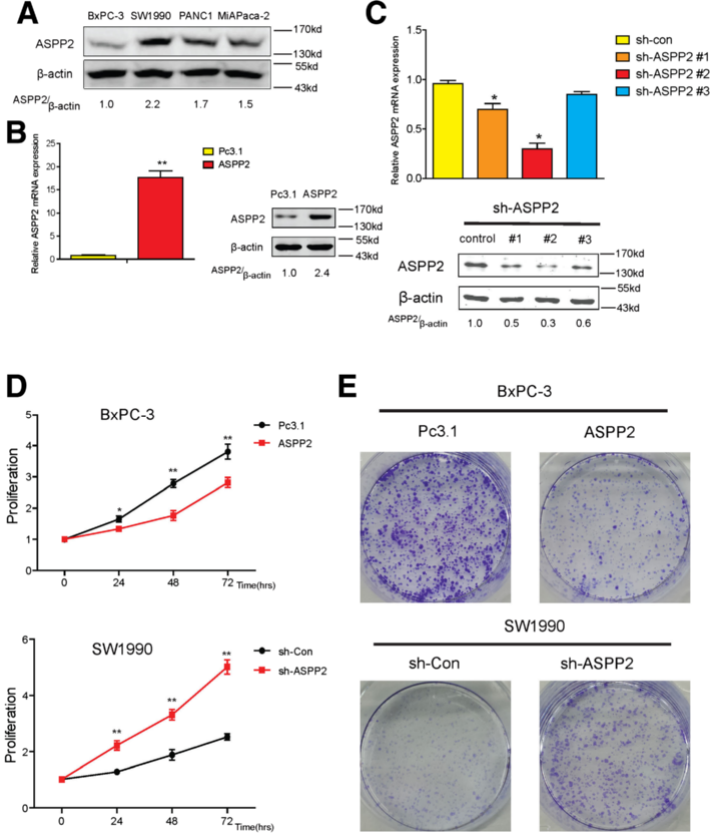
ASPP2 inhibits proliferation of pancreatic cancer cell. **a** The expression of ASPP2 in different pancreatic cancer cell lines were shown. **b** The mRNA and protein expression of exogenous ASPP2 in BxPC-3 cells with stable ASPP2. **c** The mRNA and protein expression of ASPP2 in SW1990 cells which were stably knockdown ASPP2. **d** CCK-8 assays were performed in BxPC-3 cells with stable ASPP2 and SW1990 cells with ASPP2 stably knockdown in the indicated time. **e** Colony formation assays were performed in the cells of (D). Data represent the mean ± SD of three independent experiments (*P < 0.05, **P < 0.01)

To study the role of ASSP2 in cancer, we firstly analyzed its expression in series of pancreatic cancer cell lines using NCBI GEO database [27] and the data suggest that ASSP2 expression significantly differs in different PC cell lines. Low ASPP2 expression cell line BxPC-3 and high ASPP2 expression cell line SW1990 were used for further research (Fig. 2a). We organized ASPP2 stably over-expression cell lines in BxPC-3 and ASPP2 stably knockdown cell lines in SW1990, the mRNA and protein expression of ASPP2 were shown (Fig. 2b, c and Additional file 1: Figure S1A). The #2 of ASPP2 knockdown cell lines was used for next experiments. Forced ASPP2 over-expression resulted in a significant decrease of cancer cell proliferation, Knockdown of ASPP2 enhanced proliferative activity (Fig. 2d). Consistently, over-expression of ASPP2 attenuated cancer cell clone formation and vice versa (Fig. 2e). Together, these results reveal that down-regulation of ASPP2 could enhance cell proliferation of pancreatic cancer.

### ASPP2 negatively regulate basal autophagy in pancreatic cancer cell lines

**Fig. 3:**
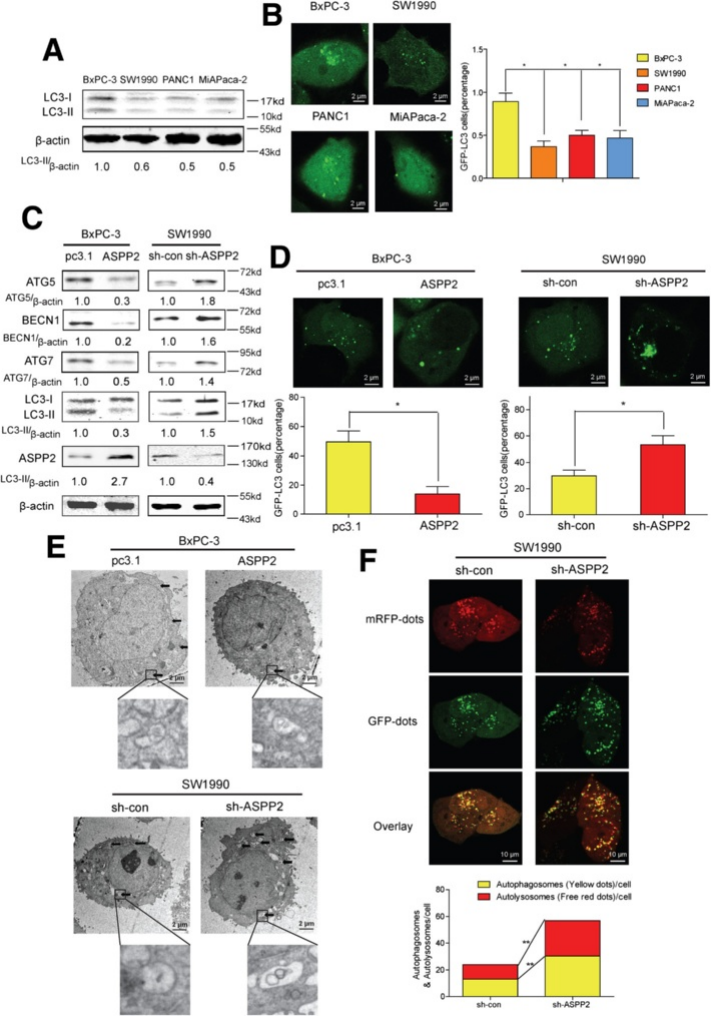
ASPP2 negatively regulate basal autophagy in pancreatic cancer cell lines. **a** Immunoblotting analysis was performed to determine LC3-II in different pancreatic cancer cell lines; **b** GFP-LC3 was transfected into PC cells with different expression levels of ASPP2 and autophagosome formations was observed under microscopy. Representative picture and the percentage of autophagic cells were calculated in 5 random fields. **c** Western blot assay was performed to determine the expression of autophagy-related protein in BxPC-3 cells with stable ASPP2 and SW1990 cells with ASPP2 stably knockdown. **d** The cells in (C) were transfected with GFP-LC3 expression plasmids and autophagosome formations was observed under microscopy. **e** Representative electron micrographs of autophagic vesicles were shown. Arrows denote autophagosomes. Magnified images also were shown. **f** Representative images of LC3 staining in sh-con/sh-ASPP2 SW1990 cells infected with adenovirus-delivering mRFP-GFP-LC3. Quantification of autophagosome and autolysosome formation represents punctures staining sites per cell of 5 independent images. Data represent the mean ± SD of three independent experiments (*P < 0.05, **P < 0.01)

Recently, two groups found that ASPP2 could regulate survival autophagy and autophagic apoptosis through different ways in hepatoma cells,[21, 22] which indicated that ASPP2 could be the Indispensable regulator in autophagy. In pancreatic cancer, autophagy play essential function in cell growth under basal condition.[28] To examine its regulation on basal autophagy, firstly, we analyzed the autophagic activities of pancreatic cancer cell lines. Unexpectedly, BxPC-3 cells with low ASPP2 expression expressed higher LC3-II, the marker of autophagosomes,[29] sw1990 cells with high ASPP2 expression formatted less LC3-II (Fig. 3a). For directly observing the autopahgy process, we also transfected GFP-LC3 into these four PC cell lines and analyze the formation of autophagosomes,[29] we found that more autophagosomes were formed in the BxPC-3 cells than SW1990, PANC1and MiAPaca-2 cells (Fig. 3b). All the data presented suggest that autophagy negatively correlated with the expression of ASPP2.

Based on these data, we hypothesized that ASSP2 can negatively regulate basal autophagy in pancreatic cancer cells. To test this hypothesis, we checked the Autophagy-Related Gene Family in BxPC-3 cells with ASPP2 overexpression and in SW1990 cells with ASPP2 knockdown. Overexpression of ASPP2 could significantly decrease the amount of LC3-II and ATG family expression, and knockdown of ASPP2 enhanced it (Fig. 3c). With GFP-LC3 system, we confirmed the regulation of ASPP2 in autophagy (Fig. 3d). In addition, as presented in Fig. 3e, the accumulation of autophagic vacuoles was dramatically decreased in ASPP2 overexpression BxPC3 cells and silencing of ASPP2 reversed it. Furthermore, for detecting the autophagic flux, we examined mRFP-GFP-LC3 puncta formation with fluorescence microscope. Consistently, ASPP2 decreased the autophagosomal-lysosomal fusion process, which also supports ASPP2 as the negative regulator for autophagy under basal condition (Fig. 3f). As a P53 interacting-protein,ASPP2 often regulate cell function in P53-dependent way. P53 was also reported as the core-regulator in autophagy.[30] to exclude the function of P53 in the regulation of autophagy, we also check the basal autophagy with P53 inhibitor, pifithrin-α [31]. However, inhibition of P53 could not reverse the function of ASPP2 in autophagy, which suggested that ASPP2 regulated basal autophagy in P53-indepdent way (Additional file 1: Figure S1B).

### ASPP2 regulate autophagy through AMPK-mTOR pathways

**Fig. 4:**
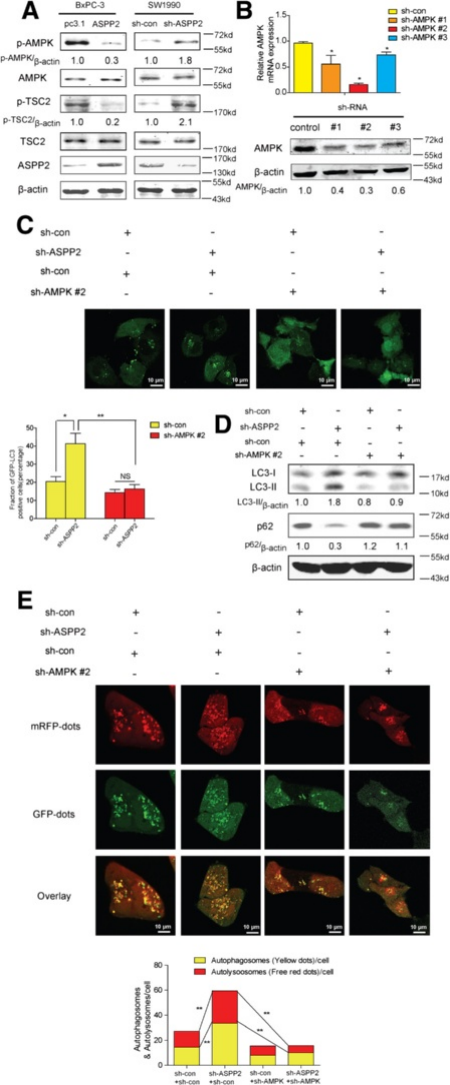
ASPP2 regulates autophagy through AMPK-mTOR pathways. **a** Indicated proteins were detected with immunoblots in BxPC-3 cells with stable ASPP2 and SW1990 cells with ASPP2 stably knockdown. **b** Identification of shRNA activity of AMPK with RT-PCR and immunoblots. **c** sh-con/sh-ASPP2 SW1990 cells with GFP-LC3 were transfected with sh-Con/sh-AMPK, autophagosomes formation was observed under microscopy. The percentage of autophagic cells was calculated in 5 random fields. **d** Indicated molecules were detected with immunoblots in sh-con/sh-ASPP2 SW1990 cells transfected with sh-Con/sh-AMPK. **e** sh-con/sh-ASPP2 SW1990 cells were transfected with sh-Con/sh-AMPK, then infected with adenovirus-delivering mRFP-GFP-LC3. Quantification of autophagosome and autolysosome formation represents punctures staining sites per cell of 5 independent images. Data represent the mean ± SD of three independent experiments (*P < 0.05, **P < 0.01)

AMPK is a key cellular energy sensor and functions to maintain energy homeostasis upon nutrient starvation,[32] it could regulate autophagy through mTOR pathway.[33] Firstly, we checked the phosphorylation of AMPK and TSC2. Down-regulation of ASPP2 could increase the phosphorylation of AMPK and TSC2 and vice versa (Fig. 4a). For confirming the ASPP2 regulate autophagy through AMPK, we knockdown the AMPK expression with shRNA (Fig. 4b),and found that AMPK knockdown could significantly inhibit the autophagic flux upregulation induced by ASPP2 knockdown (Fig. 4c,d,e). These results indicate that ASPP2 regulate autophagy through AMPK-mTOR pathways.

### ASPP2 can facilitate gemcitabine induced cell apoptosis through regulation of autophagy in vitro and in vivo

**Fig. 5:**
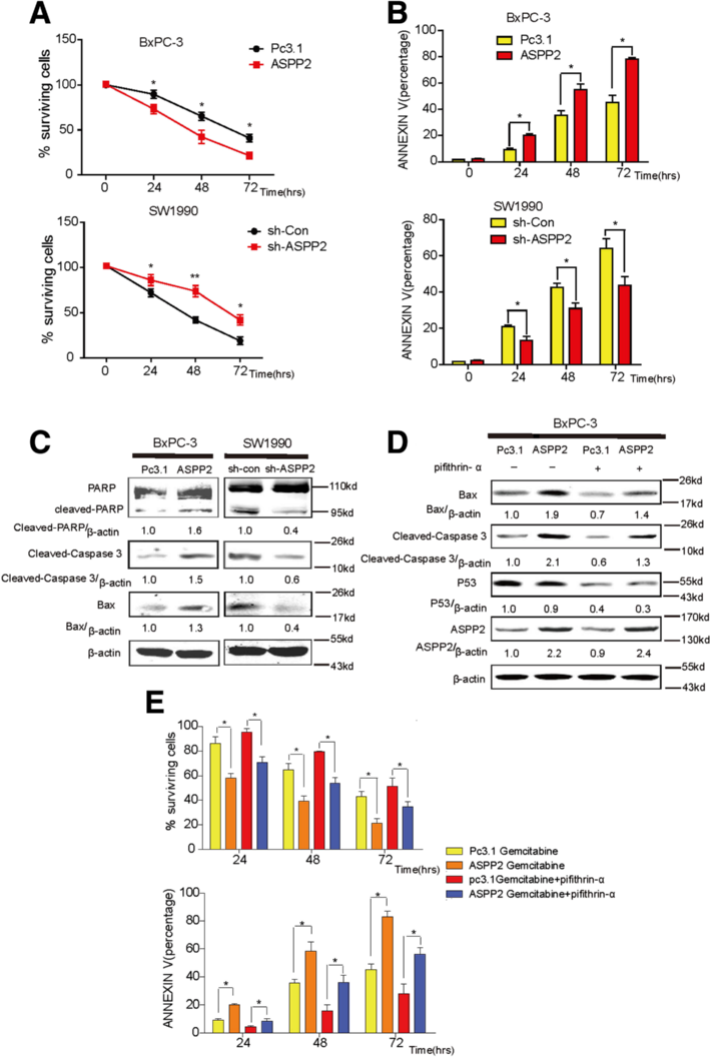
ASPP2 can enhance gemcitabine induced cell apoptosis. **a** The surviving rate of cancer cells were detected with CCK-8 assays in BxPC-3 cells with stable ASPP2 and SW1990 cells with ASPP2 stably knockdown treated with gemcitabine (1 μM) for indicated times.**b** the rate of apoptosis cells were determine by Annexin V-FITC Apoptosis Detection Kit in cells of (A). **c** The indicated proteins were detected with immunoblots in BxPC-3 cells with stable ASPP2 and SW1990 cells with ASPP2 stably knockdown treated with gemcitabine (1 μM) for 48 h. **d**,**e** the indicated molecules, The surviving rate of cancer cells and the rate of apoptosis cells were detected in BxPC-3 cells with stable ASPP2 treated with gemcitabine (1 μM) or gemcitabine (1 μM) plus pifithrin-α (10 μM). Data represent the mean ± SD of three independent experiments (*P < 0.05, **P < 0.01)

Gemcitabine is currently the standard chemotherapy treatment at all stages of pancreatic cancer.[34] Survival benefit and clinical impact however remain moderate due to a high degree of drug resistance. To dissect the effect of ASPP2 on gemcitabine mediated cell apoptosis, we treated these cells with gemcitabine. As shown in Fig. 5a, overexpression of ASPP2 can promote the gemcitabine induced cell death and knockdown of ASPP2 could inhibit it. To further define the role of ASPP2 in determining the sensitivity of PC to gemcitabine, we checked the cell apoptosis induced by gemcitabine, which is consistently with the cell death results (Fig. 5b). Apoptosis protein expression also proved it (Fig. 5c). All the results suggest that ASPP2 expression level can determine the susceptibility of the cells to gemcitabine treatment.

**Fig. 6:**
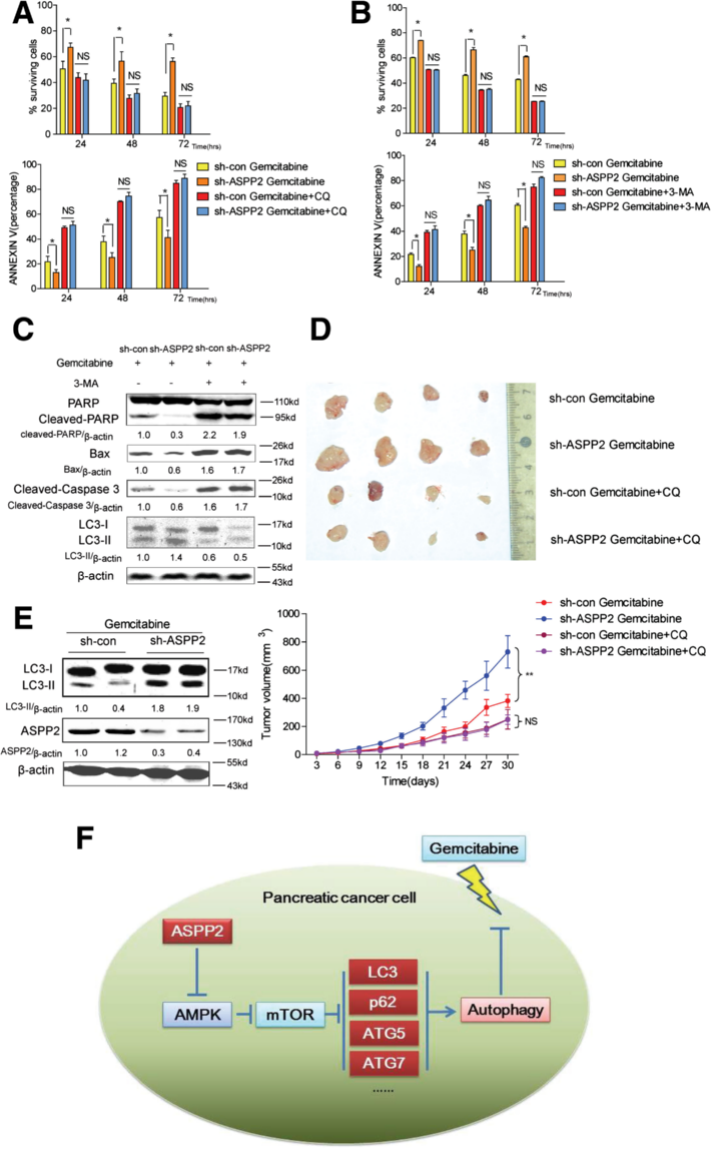
ASPP2 facilitate gemcitabine induced cell apoptosis through regulation of autophagy in vitro and vivo. **a**–**b** SW1990 cells with ASPP2 stably knockdown treated with gemcitabine (1 μM) combined with/without CQ (10 μM)/3-MA (10 mM) for indicated times. The rate of surviving and apoptosis cells was detected. **c** The indicated proteins were detected with immunoblots in SW1990 cells with ASPP2 stably knockdown treated with gemcitabine (1 μM) with or without 3-MA (10 mM) for 48 h. **d** SW1990 cells with ASPP2 stably knockdown were injected into nude mice; gemcitabine combined with CQ or not were injected peritumorally. Tumors volumes were determined as the indicated time point. The expression of autophagy marker LC3 and ASPP2 were also shown in (**e**). **f** A model for regulation of autophagy by ASPP2 in pancreatic cancer. Down-regulation of ASPP2 in pancreatic cancer promotes autophagy through activates AMPK-mTOR pathways, resist to gemcitabine induced apoptosis. Data represent the mean ± SD of three independent experiments (*P < 0.05, **P < 0.01)

As ASPP2 could interact with P53 to Specifically Stimulate the Apoptotic Function of p53, we firstly checked that whether ASPP2 regulated gemcitabine-resistance in pancreatic cancer. With pifithrin-α treatment, we found that inhibition of P53 could not reverse the ASPP2-inudced cell apoptosis, which suggested that ASPP2 regulated gemcitabine-induced cell death in P53-indepdent way (Fig. 5d,e). As shown previously, ASPP2 can negatively regulate autophagic activity, so we next exam whether ASPP2 determining the susceptibility of cells to gemcitabine is dependent on its regulation of autophagy. To answer this question, we treated ASPP2 shRNA transduced SW1990 cells with gemcitabine in the presence or absence of CQ and 3-MA, which are the widely used autophagy inhibitors, and cell death and cell apoptosis were analyzed. The protection of knockdown of ASPP2 in SW1990 cells from gemcitabine induced apoptosis was dramatically impaired in the presence of autophagy inhibitors, suggesting that ASPP2 regulate gemcitabine induced cell apoptosis via autophagy (Fig. 6a, b, c). To dissect the ASPP2 role in regulation of gemcitabine induced cell apoptosis *in vivo*, we inoculated SW1990 cells with ASPP2 knockdown into nude mice and determine the tumor size at indicated time point post-inoculation. As shown in Fig. 6d and e, ASPP2 knockdown cells were more resistant to gemcitabine treatment; however, this is significantly impaired following treatment of CQ, further establishing the essential role of autophagy in determining the resistance to gemcitabine *in vivo*. Collectively, all the data presented here demonstrate that decrease expression of ASPP2 can confer cell the insensitivity to gemcitabine, which is dependent on its activation of autophagy.

## Discussion

The capability of cancer cells to escape the cytotoxic effect of chemotherapeutic drug may result from genetic mutations that affect cell cycle, apoptosis or accumulation of drugs inside of the cell.[35, 36] In this study, we revealed the mechanisms by which pancreatic cancer cells to escape chemotherapeutic drug (gemcitabine) mediated cell apoptosis via decrease of ASPP2 expression to activate the autophagic activity.

We found that ASPP2 expression level was preferentially down-regulated in cancerous tissues compared with para-cancerous tissues from the pancreatic cancer patients. ASPP2 expression levels were different in distinct PC cell lines and ASPP2 could negatively regulated cancer cell proliferation. We found that the expression levels of ASPP2 negatively associated with the autophagic activities in the pancreatic cancer cell lines. Through overexpression and knockdown of ASPP2, we demonstrated that ASPP2 can negatively regulate autophagic activity. Furthermore, we demonstrated that ASPP2 regulated autophagy through AMPK-mTOR pathways. We also found that ASPP2 could promote the gemcitabine induced cell apoptosis in pancreatic cancer cells, and knockdown ASPP2 can enable the phenotype of the cells to resistant for gemcitabine treatment. In addition, the effect of protecting cells from gemcitabine treatment through knockdown of ASPP2 was significantly compromised by repression of autophagy in vitro and in vivo, indicating that ASPP2 determining the sensitivity of pancreatic cancer cells to gemcitabine was dependent on its regulation of autophagy (Fig. 6f).

Autophagy is the ubiquitous cellular process that involves cell degradation of unnecessary or dysfunctional cellular components through the lysosomal machinery; it plays a critical role in removing protein aggregates, as well as damaged or excess organelles, to maintain intracellular homeostasis [37]. Autophagy is widely involved in many physiological process, such as cell survival, embryo development, longevity, clearance of toxic aggregate-prone proteins, cell surface receptor trafficking and protection of host from pathogen infection.[19, 38–40] Dysregulation of autophagy contributes to a number of diseases including tumorigenesis.[17, 19] However, the role of autophagy in cancer is still controversial. It was reported that inhibition of autophagy can increase chemotherapy-induced apoptosis in hepatocarcinoma cells,[41] colon cancer,[42] and esophageal cancer cells.[43] In our study, we found that the decrease of ASPP2 could enhance the autophagic activity of pancreatic cancer cells, and this lead to pancreatic cancer cells less sensitive to gemcitabine induced cell apoptosis *in vitro* and *in vivo*; Lower expression of ASPP2 was also correlated with poor outcome of gemcitabine treatment and survival rates. We also found that ASPP2 was down-regulated in the pancreatic cancer tissues compared with para-pancreatic cancer tissues, suggesting that decrease of ASPP2 leading to upregulated autophagy might serve as a chemotherapy intrinsic defense mechanism for pancreatic cancer cells.

## Conclusions

Taken together, the data provide new insights into the mechanisms by which decrease of ASPP2 in pancreatic cancer cells can interfere with the effectiveness of chemotherapy via enhanced autophagy. These results reveal ASPP2 as a crucial and unexpected switch for the sensitivity to gemcitabine phenotype of pancreatic cancer via regulation of autophagy, which suggests that in ASPP2 low expression patients gemcitabine combined autophagy inhibitors could significantly promote cancer cell apoptosis. Our data identify a molecular link between aberrant ASPP2 expression in pancreatic cancer and susceptibility to gemcitabine treatment. A better understanding of this process may lead us to new methods to overcome drug resistance in this aggressive disease.

## Methods

### Patients and samples

Twelve cancer tissues were (for qRT-PCR and WB) from the patients which receiving curative resection in Changhai Hospital, Shanghai, China from January 2013 to January 2014. 65 pancreatic cancer tissues (for IHC) were randomly retrieved from pancreatic cancer patients receiving curative resection in Changhai Hospital, Shanghai, China from January 2008 to January 2010 (see detailed clinical pathologic features in Table 1). All patients were followed up until January 2013, with a median observation time of 21 months. Matched pairs of primary pancreatic cancer samples and adjacent pancreatic tissues were used for analysis of ASPP2 expression. Participants provide their written informed consent to participate in this study, and this study was performed in according to an established protocol approved by the Ethic Committee of Changhai Hospital.

### Cell culture and reagent

Human pancreatic cancer cell lines BxPc-3, SW1990, Panc-1 and MiaPaCa-2 were purchased from Cell Bank of Type Culture Collection of Chinese Academy of Sciences. They were cultured in 10 % FBS DMEM/RPMI1640 at 37 °C and 5 % CO2. Autophagy inhibitors, 3-MA and chloroquine were purchased from Sigma (San Diego, CA). ASPP2 antibody (sc-53861) is mouse monoclonal IgG1 against amino acids 691–1128 of ASPP2 of human origin. The following antibodies were used for Western blot: AMPK, p-AMPK, Actin (Santa Cruz), p-TSC2, TSC2, Atg5, Atg7, Beclin1, p62, LC3 (Cell signal technology).

### RT-qPCR assay

Total RNA was extracted by using Trizol reagent (Invitrogen, Carlsbad, CA), and the reverse-transcription reactions were performed using an M-MLV Reverse Transcriptase kit (Invitrogen). Real-time PCR was performed using a standard SYBR Green PCR kit (Toyobo, Osaka, Japan). The primers used in RT-PCR as Followed. mRNA levels are calculated as fold change of control.

### Sequence of primers for real-time PCR

**Table:** 

Primer	Sequence (5′ to 3’)
ASPP2 forward primer	5’-TAAGCAATGGGAAACTTGTGG-3’
ASPP2 reverse primer	5’-CATCCGAGGCATAGTAGACGA-3’
18 s forward primer	5’-CGGCTACCACATCCAAGGAA-3’
18 s reverse primer	5’-GCTGGAATTACCGCGGCT-3’

### ShRNA Interference

We generated plasmid vectors encoding shRNAs targeting ASPP2/AMPK or scramble shRNA using pENTR™/U6 expression vector (Invitrogen, Carlsbad, CA), and designated them as sh-Con and sh-ASPP2/sh-AMPK, respectively. The synthesized oligonucleotides were as follows:

**Table:** 

Gene	Forward	Reverse
ASPP2#1	5’-CACCGCAGAATGCCAAGCTACAACACGAATGTTGTAGCTTGGCATTCTGC -3’	5’-AAAAGCAGAATGCCAAGCTACAACATTCGTGTTGTAGCTTGGCATTCTGC -3’
ASPP2#2	5’-CACCGCTGCAGTAGGTCCCTATATCCGAAGATATAGGGACCTACTGCAGC -3’	5’-AAAAGCTGCAGTAGGTCCCTATATCTTCGGATATAGGGACCTACTGCAGC -3’
ASPP2#3	5’-CACCGCGTCCGTTCTCAATGTTTGACGAATCAAACATTGAGAACGGACGC -3’	5’-AAAAGCGTCCGTTCTCAATGTTTGATTCGTCAAACATTGAGAACGGACGC -3’
AMPK#1	5’-CACCGCTTGATGCACACATGAATGCCGAAGCATTCATGTGTGCATCAAGC -3’	5’-AAAAGCTTGATGCACACATGAATGCTTCGGCATTCATGTGTGCATCAAGC -3’
AMPK#2	5’-CACCGCAGGCCCAGAGGTAGATATACGAATATATCTACCTCTGGGCCTGC -3’	5’-AAAAGCAGGCCCAGAGGTAGATATATTCGTATATCTACCTCTGGGCCTGC -3’
AMPK#3	5’-CACCGCAGAAGTATGTAGAGCAATCCGAAGATTGCTCTACATACTTCTGC -3’	5’-AAAAGCAGAAGTATGTAGAGCAATCTTCGGATTGCTCTACATACTTCTGC -3’

### MTT assay

The cancer cells were seeded in 100 μl growth medium including 5 × 103 cells per well in 96-well plates. Cells were treated with indicated regents or not. Every 24 h until 72 h, CCK-8 solution was added to wells and incubation at 37 °C for 2 h. Cell viability was measured. Viability is given as a percent of the control value.

### Colony formation assay

Five hundred cancer cells per well were seeds in 6-wells plate. After cultured for 2–3 weeks, we terminate cell culture and wash the plate with PBS for two times, fixed cells with 4 % Paraformaldehyde for 15 min, Incubate cells with trypan blue for 10 min and wash the staining solution.

### Apoptosis study

The Annexin V-FITC Apoptosis Detection Kit was purchased from eBioscience, Inc. (San Diego, CA) and used as recommended by manufacturer’s instruction.

### Western blotting

Western blot analysis was carried out on 10 % SDS–PAGE. Briefly, proteins were electrotransferred onto nitrocellulose filter. After blocking for 1 h in PBS with 0.1 % Tween 20 (PBST) and 5 % BSA, the membranes were incubated over night with specified primary antibody in PBST containing 5 % BSA. Detection was carried out by the use of HRP conjugated IgG assay kit (Sigma, St. Louis, MO). The former band was stripped with Stripping buffer (P0025, Beyotime) and incubated with other antibodies. the relative density of WB bands with ImageJ2X.

### Transmission electron microscopy

Cells were fixed for 1 h at 4 °C in 1.6 % glutaraldehyde in 0.1 M Sörensen phosphate buffer (pH 7.3), washed, fixed again in aqueous 2 % osmium tetroxide and finally embedded in Epon. Electron microscopy was performed with a JEM-2000 EX transmission electron microscope, on ultrathin sections (45 nm) stained with lead citrate and uranyl acetate.

### Immunohistochemistry (IHC)

Immunohistochemistry was performed using EnVisiontm system in according to the manufacture’s instruction. The antibody against ASPP2 (Santa Cruz) was tested on sections from formalin-fixed paraffin-embedded pancreatic cancer and para-cancerous tissue samples. Images were obtained with a Zeiss AXIO Imager. A1 microscope (Zeiss, Jena, Germany) equipped with an AxioCam (Zeiss) and the AxioVision 4.6 software (Zeiss). The density of ASPP2 positive staining was measured by a computerized image system (Leica Microsystems Imaging Solutions Ltd, Cambridge, United Kingdom). The patients were divided to two groups (“low ASPP” and “high ASPP”) according to median of ASPP2 expression in cancer samples.

### Tumor xenograft study

All animal experimentation proceeds according to the Standard of IACUC (Institutional Animal Care and Use Committee) and performs in according to an established protocol approved by the Ethic Committee of Changhai Hospital. 2 × 10^6^ cells Cancer cells were introduced by subcutaneous implantation of in 10 % FBS DMEM into 6-week-old immunodeficient nude mice. 1 week later, Gemcitabine (40 mg/kg) with or without Chloroquine (CQ, 45 mg/kg) was injected every 2 days into tumors. The mice were sacrificed 5 weeks after tumor implantation. The volumes of tumors were measured and calculated as V = a x b^2^ x π/6.

## Additional file

Additional file 1: Figure S1. (A) The expression of ASPP2 was detected in BxPC-3 cells with stable ASSP2 over-expression and SW1990 cells with ASPP2 knockdown. (B) Indicated molecules were measured with immunoblots in BxPC-3 cells with stable ASSP2 over-expression treated with pifithrin-α (10 μM). (JPEG 139 kb)

## Abbreviations

ASPP2: Apoptosis-stimulating of p53 protein 2
RT-qPCR: Reverse Transcription Quantitative Polymerase Chain Reaction
shRNA: Short hairpin RNA
AMPK: Adenosine 5’-monophosphate (AMP)-activated protein kinase
PC: Pancreatic cancer
ATG: Autophagy-related
GFP: Green Fluorescent Protein
RFP: Red fluorescent protein
CQ: Chloroquine
