# Supplementary figures and images for: Downregulation of ASPP2 in pancreatic cancer cells contributes to increased resistance to gemcitabine through autophagy activation
Source: Mol Cancer. 2015 Oct 5;14:177. doi: 10.1186/s12943-015-0447-5 (PMC4594892; doi:10.1186/s12943-015-0447-5)

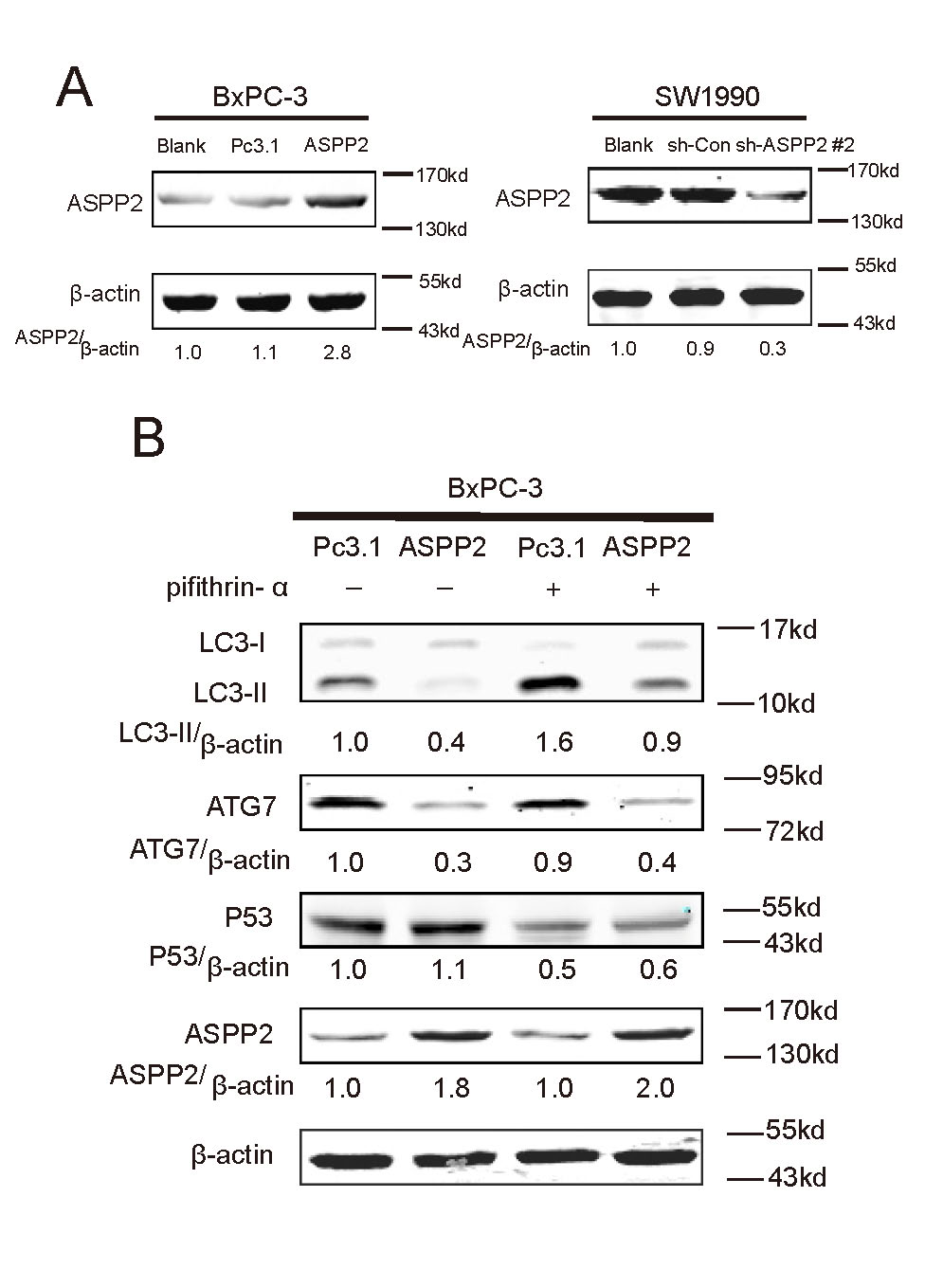

Supplement: Additional file 1: Figure S1. — (A) The expression of ASPP2 was detected in BxPC-3 cells with stable ASSP2 over-expression and SW1990 cells with ASPP2 knockdown. (B) Indicated molecules were measured with immunoblots in BxPC-3 cells with stable ASSP2 over-expression treated with pifithrin-α (10 μM). (JPEG 139 kb) [file 12943_2015_447_MOESM1_ESM.jpg]
